# Aesthetic reconstruction of the severely disfigured burned face: a creative strategy for a “natural” appearance using pre-patterned autogenous free flaps

**DOI:** 10.1186/s41038-015-0014-8

**Published:** 2015-09-27

**Authors:** Elliott H. Rose

**Affiliations:** Division of Plastic & Reconstructive Surgery, The Mount Sinai Medical Center, 895 Park Avenue, New York, NY 10075 USA

**Keywords:** Facial burns, Facial reconstruction, Autogenous reconstruction, Microvascular free flaps, Burn reconstruction, Pre-patterned flaps, Scapular flaps, Pedicled forehead flaps, Radial forearm flap, Fascia lata slings, Temporoparietal flaps

## Abstract

The author reviews his pioneering work in aesthetic restoration of the severely disfigured burn face first introduced in 1995 and refined over the past two decades. The reader will be exposed to the step by step approach to achieving cosmetic enhancement and functional rehabilitation of advanced facial burns. The “keystone” of the autogenous reconstruction is the *pre-patterned*, *sculpted microvascular free flap* designed to fit like the “piece of a puzzle” into the aesthetic units of the face to replace disfiguring burn scars. Aggressive intraoperative “sculpting” is employed both “*in situ*” at the donor site and during the flap transfer to simulate the normal facial contours and planes. Comparisons of the author’s approach are made to the whole spectrum of reconstructive modalities ranging from conventional grafting to expanded pre-fabricated flaps and even to CTA face transplants; advantages/disadvantages of each are discussed. The pre-patterned, sculpted microvascular (MV) free flap offers the benefit of a *single-stage* transfer of composite skin/soft tissue hiding the seams at the junction of facial planes. When harvested from distant donor sites, the donor deformities can easily be concealed. The MV free tissue transfer offers the substrate that can be sculpted into nuanced facial components as well as the “palette” upon which the face can be painted with creative camouflage makeup. The soft contour and texture of the autogenous patterned transfers translates into a “natural” facial appearance while preserving fluid motions of facial expression.

## Introduction

The severely disfigured burned face offers a unique challenge to the reconstructive surgeon. The complexities of thick hypertrophic plaques of scar, ambiguities on facial planes, functional burn contractures, architectural distortions and aesthetic disfigurement compel excellence in planning and execution of the restorative process. Less extensive procedures such as Z-plasties, local flaps (i.e., transposition [1], propeller flaps [2], square flap [3]) and full thickness skin grafts are useful in addressing more limited functional concerns such as nostril stenosis, lip or lid ectropion, exposed ear cartilage, and perioral contractures [4]. The more demanding challenge, however, is the extensive facial burn crossing multiple aesthetic units and requiring large areas of coverage in replacement of the disfiguring hypertrophic/keloid scars. Feldman has described “megaunits” of thick split-thickness grafts [5] to resurface facial aesthetic units (initially described by Gonzales-Ulloa) [6], but, in my experience, these grafts yield “flat, inanimate facies” devoid of character and facial expression.

## Review

### Limitations of conventional reconstruction

Disfiguring burns of the face are often characterized by misalignment of key facial structures particularly in the central face (nose, lips, and eyelids), distortion of facial planes, and restricted facial movement and expression. Extensive burns often leave little adjacent skin for local/region flap transpositions. Traditional skin grafting often yields a corrugated, “woody texture” to the grafted skin as well as a poor color match and indistinct facial planes. Movement is compromised given the face a “flat,” artificial look with limited animation.

### Expanded flaps, pre-fabricated flaps and super-thin flaps

In my recent travels to China, I was impressed by the use of “mega” expanded blocks of composite skin pre-fabricated *in adjacent areas* (*shoulder*, *supraclavicular*) *or at distant site* (i.e., *lateral chest wall*)*.* While I was lecturing at the 10th People’s Hospital in Shanghai in May 2014, Professor Yixin Zhang showed me elegant patient examples of expanded skin with sufficient surface area to cover the entire face. Many well-crafted papers, of course, have been written in the Asian and world literature describing “super-thin” flaps [7–9], perforator super charged subdermal vascular network flaps [10], and *pre-fabricated* expanded flaps [11] in resurfacing extensive areas of facial burns. Results are quite striking! Many of these cases, however, employ *pre-expansion* at the donor site along with the requisite lengthy amount of time to achieve adequate surface area expansion for coverage. Patients, by nature, must be exceptionally compliant and willing to accept the presence of a “melon size” balloon often in conspicuous body areas, not to mention the risk of exposure, infection, extrusion of the long-term tissue expander. Moreover, extensive scarring at the donor sites in visible area of the upper chest, shoulder, or supraclavicular regions is often very conspicuous and deforming.

### Author’s approach

In my experience, most Western patients do not have the temperament for long-term placement of a “mega” tissue expander nor the fortitude to withstand the “embarrassment” of the visible expansion balloon; rather, when given the option, will often choose the “instant gratification” offered by the *immediacy* of a free tissue transfer. In 1995, I described the application of large “pre-patterned” microvascular free flaps for resurfacing of large surface facial defects [12]. Flap design mimicked “aesthetic units” of the face thereby hiding the scars at natural seams [13]. Aggressive intraoperative “sculpting” was employed both “in situ” at the donor site and during the flap transfer to simulate the normal facial contours and planes. Over the years, these techniques have been refined and modified, but the basic principles have remained intact [14]. These select flap transfers have the *look* and *feel* of normal facial skin and provide the *palette* to sculpt the nuances of facial components and the substrate for camouflage makeup. Donor sites are routinely hidden in inconspicuous areas of the back, scalp, forehead, forearm, etc.*,* that can be covered with hair styles or clothing.

### Benefits of pre-patterned autogenous free flaps

1. One-stage transfer
2. Immediate facial *symmetry* and *balance*
3. Release of contractures
4. *Sculpted* facial planes and contours
5. *Soft* skin texture as a “palette” for corrective makeup
6. Hidden seams
7. Distant inconspicuous donor sites
8. *Natural* facial appearance
9. Fluid facial expression
10. Normal appearance at conversation distance

### Steps in author’s approach to aesthetic restoration of the burned face

1. Rebuild deep facial architecture or deep facial foundation preferably with autogenous materials (fascia lata, cartilage, bone)
2. Segmental replacement of “aesthetic” facial units with *pre-patterned MV free tissue* transfers
3. Aggressive sculpting in situ at donor site and during transfer at recipient site (Fig. 1)

**Fig. 1:**
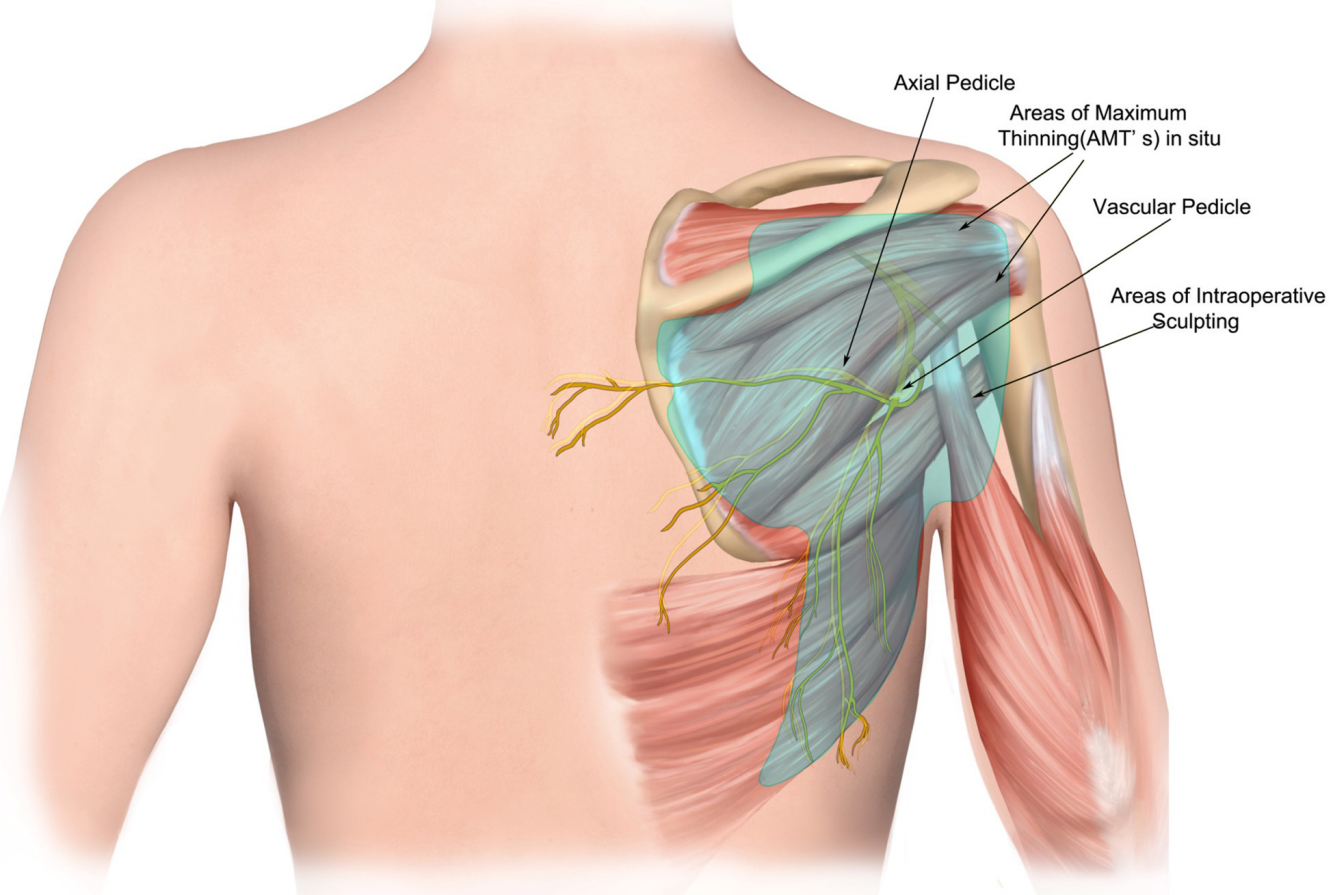
Design of pre-patterned, pre-sculpted autogenous free flap for inset into facial defects
4. Seams hidden at junction of facial planes
5. Secondary debulking, contouring of transferred units to achieve “natural” facial contour
6. Laser resurfacing of scars at periphery of the flap
7. Application of camouflage makeup to “blend” flap into facial profile

### Technique

#### Patterning of the template

The facial unit to be excised is marked at its peripheral margins with a felt tip pen. A template of the facial defect pattern is cut into a transparent film sheet (10/10 steridrape). The pattern is centered over the auscultated axial vessels or perforators at the donor site and positioned for optimal vascularity. Key landmarks are marked on the template to orient flap to the excised facial unit.

#### Donor site selection

Donor sites are chosen based on compatibility with the recipient facial unit—thickness, color match, hair density, texture, etc. My preferences are *scapula* or *DIEP flaps* for the cheek, forehead, or hemiface; *radial forearm* or scapula for neck; *temporoparietal* for ear or scalp [15]; *axial forehead* or radial forearm for nose. *Supraclavicular* perforator flaps or *submental* flaps can be used for smaller, more localized facial defects. Generally, the closer the donor flap is to the face, the closer the color match. However, after 1–2 years of flap positioning in the face, the flap often takes on the color hue of the face due to environmental exposure.

#### Flap elevation

The pre-patterned flap is thinned in situ at the donor site (except directly overlying the vascular pedicle). If perforator flaps are used, flaps can be thinned to the subdermal plexus. The flap is raised from the periphery utilizing frequent Doppler auscultation to determine depth and position of donor vessels. Flap perfusion can be ascertained by observation of capillary bleeding at the cut edges, Doppler auscultation of the pedicle within the flap, and by SPY perfusion imaging if necessary. Division of vascular pedicle to the flap is not divided until flap circulation is assured and recipient artery and vein have been isolated.

#### Facial scar excision

Only after the donor flap is raised and flap perfusion assured (see above paragraph) is the facial scar removed. The inferior third of the hypertrophic scar is elevated to allow dissection of the facial artery and vein. The pre-patterned flap is transposed to the recipient site and “tacked” at key sites to retain the orientation of the facial pattern. After the MV anastomoses have been successfully performed, the remainder of the facial scar is excised. This approach is failsafe and precludes premature creation of a huge facial defect before high probability of flap success is assured.

#### Transfer to the recipient site

The recipient vessels are exposed and the MV anastomoses carried out with minimal elevation of the keloid scar (see above paragraph). Only after patency of the vessels has been assured is the remaining keloid scar excised. If fascial suspension or architectural modifications are necessary, the slings, cartilage, or bone grafts are inset prior to flap closure. The edges of the flap are inset into the “pieces of the puzzle” and meticulously closed with deep and cutaneous stitches.

#### Sculpting

In addition to the in situ fat removal, additional “sculpting” is carried out during final inset of the flap to simulate the replaced aesthetic unit. Donor sites are closed by direct advancement or temporary STSGs. Additional refinement of the restored facial unit at 4–6 months includes additional debulking, laser resurfacing of scars, scar revision, etc. After final facial contour is achieved, patients are taught application of camouflage makeup by a professional aesthetician.

#### Donor site closure

Donor sites are closed by direct advancement and/or temporary STSGs. Dimensions of the donor site defect can usually be diminished by 50–60 % by undermining and advancement of the adjacent tissue. Temporary split skin grafts complete the closure. Residual skin grafted sites may be selectively removed by insertion of skin expanders as secondary procedures, leaving only curvilinear scars at the donor site.

### Case examples

#### Case 1

**Fig. 2:**
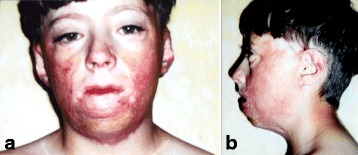
Case 1. A 12-year-old Irish boy with near total facial burns. **a** Pre-op frontal. Dense keloid scars on both cheeks, lower lip, chin, neck, and jawline. **b** Profile. Scar contracture neck and markedly retrusive chin. (Reprinted from Rose EH. Pre-patterned, sculpted free flaps for facial burns. In: Hyakusoku H, Orgill DP, Teot L, Pribaz JJ, Ogawa R (eds). *Color Atlas of Burn Surgery*. Heidelberg: Springer; 2010)

**Fig. 3:**
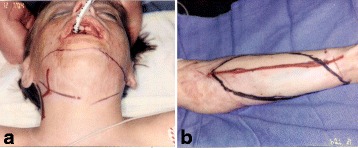
Case 1. **a** Intraoperative keloid resection of neck aesthetic unit. **b** Design of patterned radial forearm flap. (Reprinted from Rose EH. Pre-patterned, sculpted free flaps for facial burns. In: Hyakusoku H, Orgill DP, Teot L, Pribaz JJ, Ogawa R (eds). *Color Atlas of Burn Surgery*. Heidelberg: Springer; 2010)

**Fig. 4:**
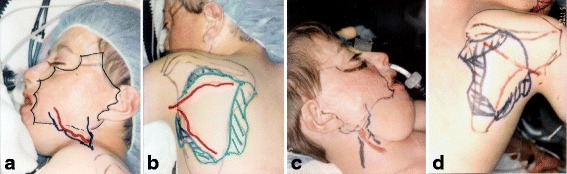
Case 1. **a** Keloid excision LT cheek unit. Doppler auscultation of facial vessels. **b** Design of pre-patterned scapular flap. *Hash marks* refer to intraoperative sculpting. Doppler auscultation of superficial circumflex scapular vessels. **c** Keloid excision RT cheek unit. **d** Design of pre-patterned scapular flap. (Reprinted from Rose EH. Pre-patterned, sculpted free flaps for facial burns. In: : Hyakusoku H, Orgill DP, Teot L, Pribaz JJ, Ogawa R (eds). *Color Atlas of Burn Surgery*. Heidelberg: Springer; 2010)

**Fig. 5:**
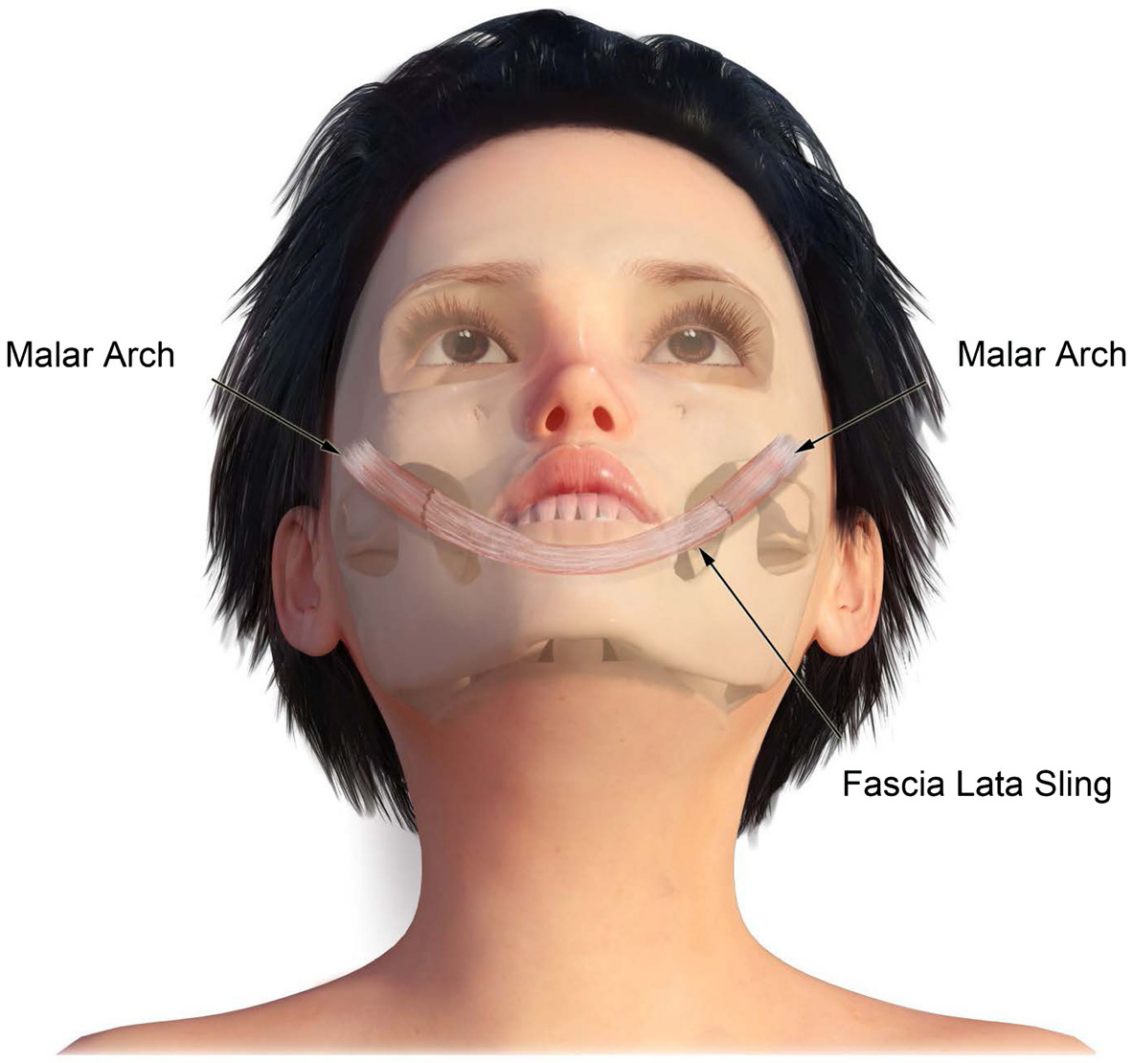
Graphic of bimalar fascia lata sling for lower lip/chin suspension

**Fig. 6:**
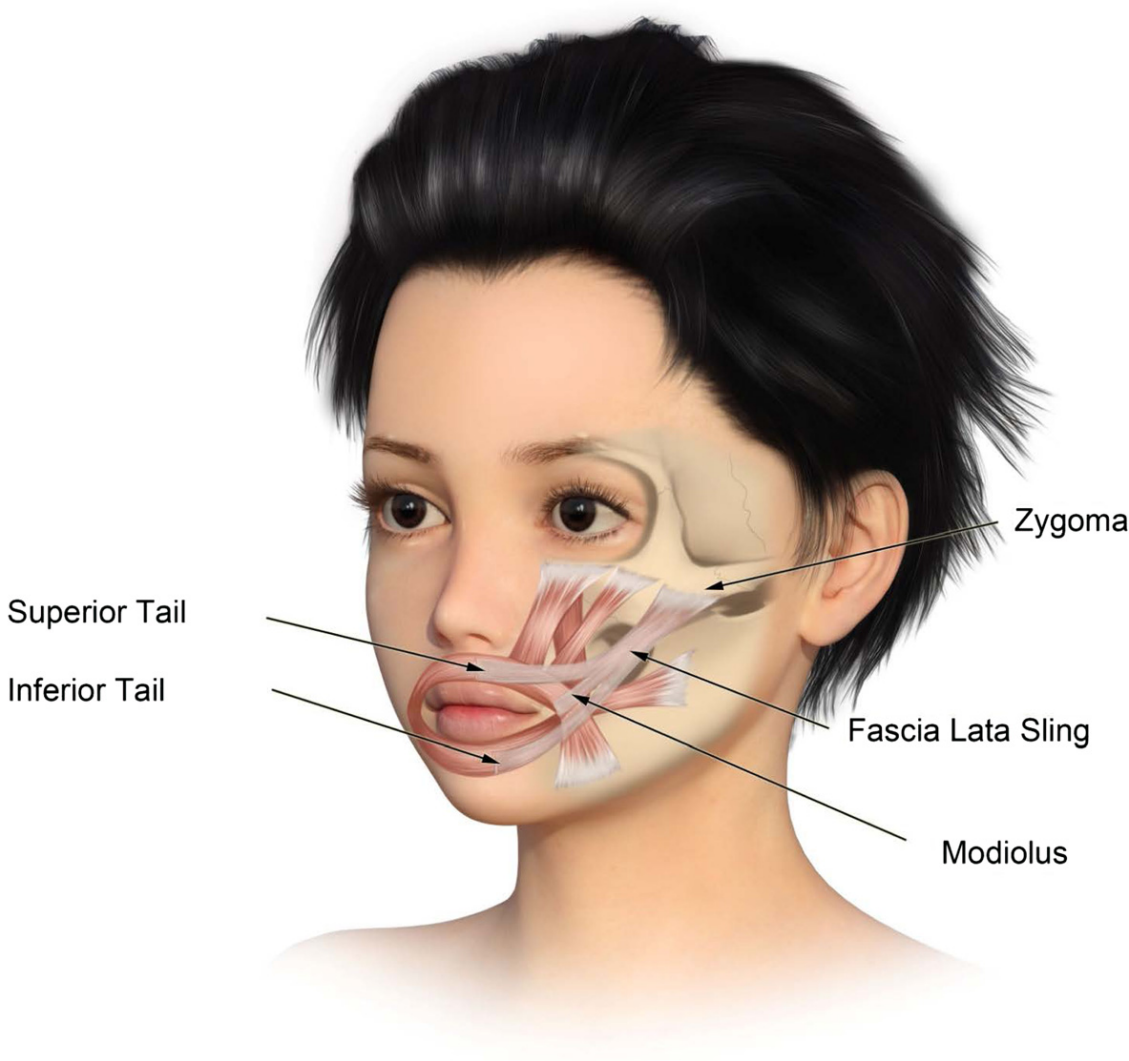
Graphic of fascia lata sling for lateral lip suspension and support of deep facial foundation

**Fig. 7:**
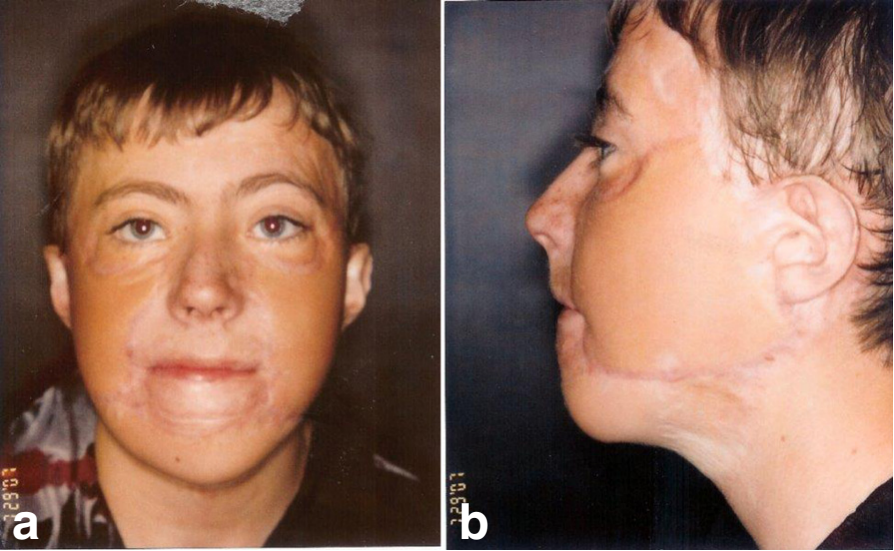
Case 1. Post-operative at 1 year after last surgery. **a** Facial contours restored with sculpted free tissue transfers. *Color hues* resemble normal facial skin. Seams hidden at junctions of aesthetic subunits. **b** Profile. Acute cervicomental angle restored. Good chin shape and projection. (Reprinted from Rose EH. Pre-patterned, sculpted free flaps for facial burns. In: Hyakusoku H, Orgill DP, Teot L, Pribaz JJ, Ogawa R (eds). *Color Atlas of Burn Surgery*. Heidelberg: Springer; 2010)

A 12-year-old Irish boy suffered a near total facial burn as a toddler when his Halloween costume caught fire. Eleven prior surgeries for correction of lip and eyelid ectropion were marginally successful. On initial exam, dense keloid scars were present over the entire face, chin, jaw, and neck (Fig. 2a). Central elements (the nose and upper lips) were predominantly spared. On profile, dense bands of contracting scar extended obliquely across the cervicomental angle causing substantial retrusiveness of the chin (Fig. 2b). The lower lip was foreshortened and evaginated with exposure of lower dentition and the dento-alveolar ridge. Multi-stage autogenous facial reconstruction was accomplished by MV free transfer of a patterned radial forearm to the neck (Fig. 3) followed by bilateral sequential patterned scapular free flaps to the right and left hemiface/cheeks, respectively (Fig. 4). Architectural modifications were coordinated with each of the flap transfers. A bimalar fascia lata sling was inset during neck reconstruction to elevate the lower lip (Fig. 5). A Porex chin implant provided the chin thrust. Fascia lata slings from the malar arches to the lateral lip modioli were placed beneath each of the scapular flaps for lateral lip support (Fig. 6). All autogenous free flaps were 100 % successful. Additional aesthetic refinements included modest debulking of the cheeks, lower lid canthoplasties, dermal plication of the nasolabial creases, and laser resurfacing of the scars. Six months after the final surgery, facial planes are restored and flaps are well incorporated into the facial geometry with seam hidden at junctions of the aesthetic units (Fig. 7a). On profile, neck contraction is mitigated; cervicomental angle is acute; and chin projection is restored (Fig. 7b).

#### Case 2

**Fig. 8:**
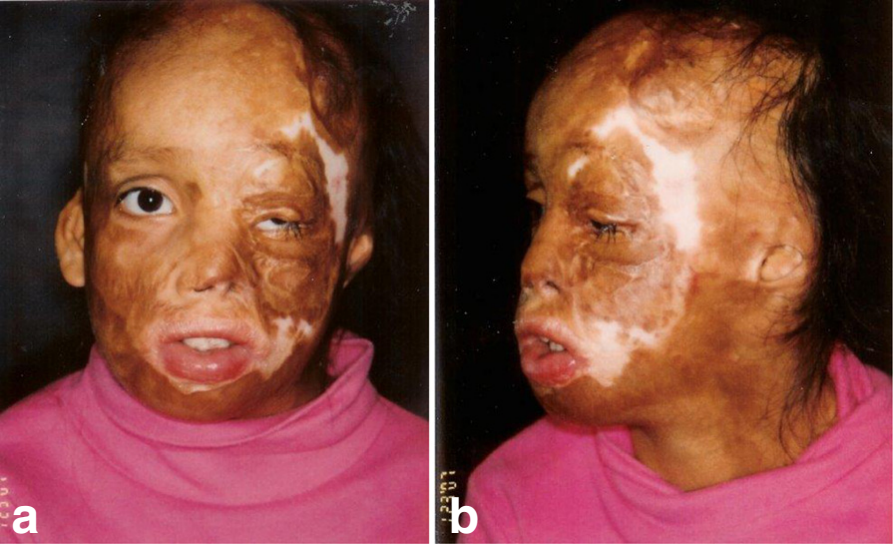
Case 2. A 10-year-old girl who suffered 80 % TBSA in crib fire as infant. **a** Frontal view. Grotesque facial scarring with distortion of facial planes, ocular displacement, nasal collapse, and microstomia. **b** Profile. Marked chin retrusion and lower lip ectropion from contracting neck scar. Deficient nasal tip and bridge projection. Large patches of scalp alopecia. (Reprinted from Rose EH. Alternative approaches to face transplantation: microsurgical approach. In: Siemienow M (ed) *The Know-How of Face Transplantation.* London: Springer; 2011

**Fig. 9:**
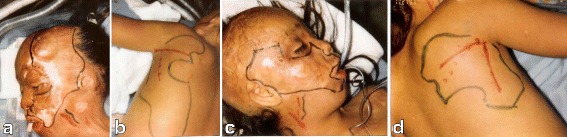
Case 2. First stage free flap transfer. **a** Keloid excision LT hemiface. **b** Design of patterned scapular flap. **c** Keloid excision RT hemiface. **d** Design of patterned scapular flap. (Reprinted from Rose EH. Alternative approaches to face transplantation: microsurgical approach. In: Siemienow M (ed) *The Know-How of Face Transplantation.* London: Springer; 2011)

**Fig. 10:**
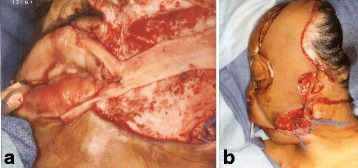
Case 2. **a** Insertion of fascia lata sling to lateral lip commissure. **b** Patterned scapular flap inset into defect. (Reprinted from Rose EH. Alternative approaches to face transplantation: microsurgical approach. In: Siemienow M (ed) *The Know-How of Face Transplantation.* London: Springer; 2011)

**Fig. 11:**
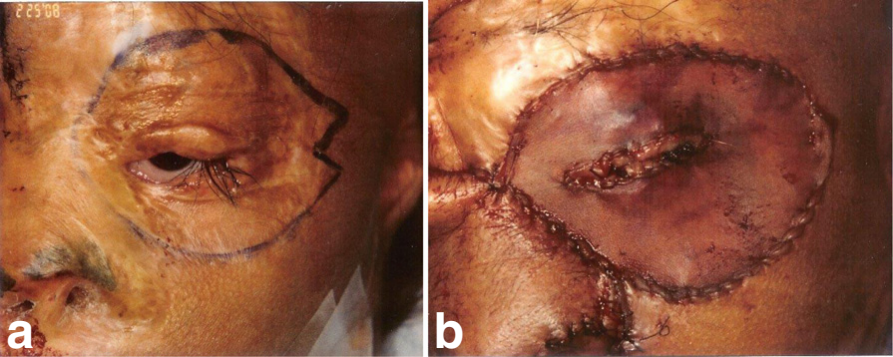
Case 2. Peri-orbital reconstruction. **a** Pattern of peri-ocular scar excision (**b**) Single sheet resurfacing with FTSG. “Slit” opening for ciliary aperture. Medial canthal ligament re-aligned with transnasal wire through glabella and lateral canthal ligament re-suspended to lateral orbital rim. (Reprinted from Rose EH. Alternative approaches to face transplantation: microsurgical approach. In: Siemienow M (ed) *The Know-How of Face Transplantation.* London: Springer; 2011

**Fig. 12:**
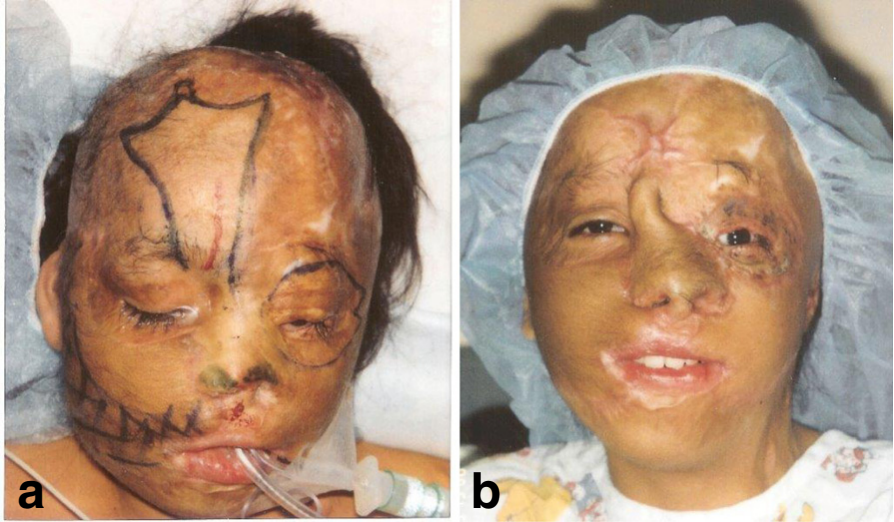
Case 2. Total nasal reconstruction. **a** Design of pedicled forehead flap centered on supratrochlear vessels. **b** Nasal flap inset. Conchal cartilage grafts used for nasal tip definition. (Reprinted from Rose EH. Alternative approaches to face transplantation: microsurgical approach. In: Siemienow M (ed) *The Know-How of Face Transplantation.* London: Springer; 2011)

**Fig. 13:**
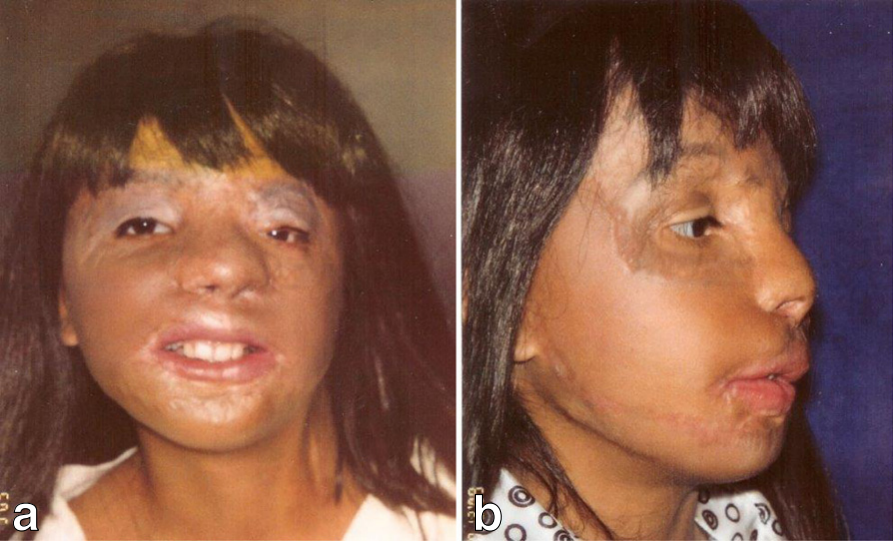
Case 2. Six months after last surgery. **a** Frontal view. Facial contours and planes restored with soft, textured surfaces. Facial components (eyes, lips, nose) in balance and symmetrical. **b** Profile. Nasal, lip, and chin projection are proportional. (Reprinted from Rose EH. Alternative approaches to face transplantation: microsurgical approach. In: Siemienow M (ed) *The Know-How of Face Transplantation.* London: Springer; 2011)

A 10-year-old girl sustained 80 % TBSA burns during a crib fire as an infant in Columbia. She was abandoned by her biological parents and brought to the United States for treatment where she was adopted by caring foster parents. Prior to treatment at The Mount Sinai Medical Center in NYC, she had undergone 10+ prior surgeries with limited success. On exam, the face was grotesquely deformed characterized by obliteration of facial planes, displacement of the LT ocular adnexae, nasal collapse, and microstomia (Fig. 8a). On profile, the chin was marked retrusive and the lower lip ectropic with exposure of the lower dentition (Fig. 8b). Nasal tip and bridge projection was deficient. Multi-stage autogenous reconstruction was initiated with sequential *pre-patterned*, *sculpted* MV *scapular flaps* to the RT and LT hemiface, respectively (Fig. 9). Deep facial foundation was restored with insertion of fascial lata slings for suspension of lateral lip commissures in conjunction with each of the scapular flaps (Fig. 10). Peri-ocular reconstruction was achieved by re-alignment of the medial canthal tendon by transnasal wire fixation and repositioning of the lateral canthal ligament to the lateral orbital rim. Both upper and lower lids were resurfaced with *single sheet* grafts to the orbital subunit with single slits opened at the ciliary apertures (Fig. 11). Total nasal reconstruction included architectural enhancement of the nasal tip with conchal cartilage grafts and dorsal resurfacing with a patterned, *pedicled forehead flap* using partial thickness burned skin (Fig. 12). The divided pedicle base was “piggy backed” to the lower eyelid for ectropion repair prior to permanent inset. Nostril patency was restored with FTSGs wrapped around nasal stents. Additional refinements included debulking/contouring of the nasal and cheek flaps, SAL, insertion of a Porex chin implant, levator advancement OS, dermal strip grafts for upper lip augmentation, nostril thinning and repositioning, scar revisions, and laser resurfacing. Six months after the final surgery, facial planes have been restored with seams hidden at junction of aesthetic units (Fig. 13a). Facial components (lips, eyes, nose) are balanced, symmetrical, and complementary. Smile is symmetrical. On profile, nasal, chin, and lip projection are proportional (Fig. 13b).

### Autogenous reconstruction vs CTA face transplant

In recent years, composite tissue allotransplantation (CTA) face transplants have been popularized by Siemienow [16], Pribaz [17], Rodriguez [18], and others to address *total* facial deformities as found in extreme burn patients. In fact, patients 1 and 2 could, in some centers, be considered candidates for a total facial transplant. In my opinion, however, in most of these cases, very acceptable facial restoration can be achieved with the application of autogenous *pre-patterned*, *pre-sculpted* MV free flaps for resurfacing of near total facial defects [19]. The one exception may be composite mid-face defects requiring multi-planar architectural reconstruction (i.e., pterygoids, tongue, palate, dentition, etc.). While these autogenous reconstructions have the disadvantage of being *multi-staged*, the need for long-term immunosuppression and the attendant morbidity (including death) is mitigated. Moreover, there are compelling financial reasons to consider multi-stage autogenous facial restoration over CTA face transplants. Preliminary data shows that while the initial operative costs incurred during facial transplants are *comparable* to conventional reconstructions of similar facial defects, post face transplantation costs are “significant and progressive and their final values indeterminable” [20].

## Conclusions

The pre-patterned microvascular free flap is the “clay” from which the shape of the face is *sculpted* and is the “keystone” of the restorative process. By replacing the aesthetic units of the facial “puzzle,” the composite flap completes the “palette” to shape the face and camouflage with creative makeup. In restoring the beauty and function of the disfigured face, the *reconstructive* and *aesthetic* disciplines are truly merged, yielding a truly natural facial appearance.

## Consent

Written informed consents were obtained from the parents (or guardians) of the 2 patients for the publication of the case report, along with all corresponding figures. Copies of the consents are available for review by the editors of this journal.
